# Acute kidney injury due to myoglobin cast nephropathy in the setting of coronavirus disease 2019-mediated rhabdomyolysis: a case report

**DOI:** 10.1186/s13256-022-03721-z

**Published:** 2022-12-28

**Authors:** Jessica J. Tuan, Onyema Ogbuagu, Deepika Kumar, Frederick Altice, Margaret Fikrig

**Affiliations:** 1 Yale University School of Medicine, Section of Infectious Diseases, 333 Cedar Street, PO Box 208022, New Haven, CT 06510 USA; 2 Yale AIDS Program, Yale University School of Medicine, 135 College Street, Suite 323, New Haven, CT 06510 USA; 3 Department of Pathology and Laboratory Medicine, Yale University School of Medicine, 333 Cedar Street, PO Box 208022, New Haven, CT 06510 USA

**Keywords:** COVID-19, SARS-CoV-2, Acute kidney injury, Rhabdomyolysis, Myoglobin cast nephropathy, Case report

## Abstract

**Background:**

We present this case of coronavirus disease 2019-associated acute kidney injury with rhabdomyolysis—with noteworthy renal biopsy findings demonstrating myoglobin cast nephropathy—to add to the limited literature on coronavirus disease 2019-related acute kidney injury and rhabdomyolysis.

**Case presentation:**

A 67-year-old Caucasian man presented to our hospital with 3 weeks of malaise and decreased oral intake and several days of abnormal taste, poor appetite, decrease urine output, gastrointestinal symptoms, and myalgias, and was ultimately diagnosed with coronavirus disease 2019. His hospital course was complicated by acute kidney injury and, upon workup of his renal failure, was diagnosed with myoglobin cast nephropathy due to coronavirus disease 2019-mediated rhabdomyolysis. Ultimately, his renal function improved following hydration back to his baseline 6 weeks after his initial diagnosis of coronavirus disease 2019.

**Conclusions:**

Given our limited knowledge of manifestations of coronavirus disease 2019, it is important to have a more in-depth understanding of the spectrum of disease of coronavirus disease 2019, which can affect various organ systems, including the kidney, and the manifestations of end-organ damage associated with it. We present this case to highlight a rarely reported finding of myoglobin cast nephropathy due to coronavirus disease 2019-mediated rhabdomyolysis.

## Background

Coronavirus disease 2019 (COVID-19) may present with a wide range of clinical manifestations, affecting various organ systems, including the kidney. Acute kidney injury in patients with COVID-19 is frequently multifactorial and evidence of direct viral invasion of renal tissue as a major etiology is lacking. Myoglobin cast nephropathy causing acute kidney injury may occur due to COVID-19-associated rhabdomyolysis.

Case reports of COVID-19-related rhabdomyolysis have been described, but rarely have demonstrated associated renal biopsy histopathology findings, and we report this case to emphasize that myoglobin cast nephropathy can occur rarely due to COVID-19-mediated rhabdomyolysis and lead to severe, though reversible, renal failure.

## Case presentation

### History

The patient was a 67-year-old Caucasian man with a past medical history of low-grade, non-invasive papillary urothelial carcinoma treated by transurethral resection of the bladder tumor, atrial fibrillation on warfarin, critical aortic stenosis corrected surgically with biologic prosthetic aortic valve replacement, coronary artery disease (CAD) status post coronary artery bypass graft (CABG), and severe emphysematous chronic obstructive pulmonary disease, who presented to our hospital from home in mid-March 2021 with symptoms of malaise and decreased oral intake for 3 weeks. For several days prior to admission, he had noted an abnormal sense of taste and poor appetite. He had symptoms of generalized fatigue, myalgias, as well as nausea, vomiting, and diarrhea. Notably, he was eating and drinking very little during this timeframe, and had produced no urine for 3 days prior to admission. Due to severe fatigue that progressed to requiring a walker to ambulate, he presented to the emergency department for evaluation. He denied fevers, chills, cough, dyspnea, headache, abdominal pain, dyspnea, and extremity edema.

Of note, he lived at home with his wife, son, and daughter-in-law as well as three grandchildren who were all healthy, none of whom had COVID-19 symptoms. He was retired and worked for an aircraft manufacturer company the past 3 years prior. He had no reported family history of renal disorders.

Of note, 3 months prior, he had been hospitalized for over a month for critical aortic stenosis requiring biologic aortic valve replacement and had CABG for CAD. At that time, his course was complicated by atrial fibrillation with rapid ventricular rate during which he developed hypotension and shock, chronic obstructive pulmonary disease exacerbation, and malnutrition secondary to severe dysphagia. Remarkably, he had been discharged to a short-term rehabilitation facility and had been home for a few weeks. He had a 30 pack-year smoking history having quit 9 years prior, but otherwise denied a current history of any substance or alcohol use. Additionally, the patient had received one dose of the messenger RNA (mRNA)-1273 COVID-19 vaccine approximately 1 month prior to his presentation.

In the emergency department, he was noted to be afebrile with a temperature 97.6 °F, hypotensive to 86/44 mmHg, with a heart rate of 73 beats per minute. His oxygen saturation was 92% on room air. On examination, he was chronically ill-appearing, but in no acute distress. His oral mucosa appeared dry. Cardiovascular examination demonstrated grade III/VI systolic murmur in the left upper sternal border. Pulmonary examination demonstrated diminished breath sounds bilaterally. He had severe right lower quadrant abdominal and suprapubic tenderness to palpation, as well as severe bilateral thigh tenderness to palpation. Extremities were warm and well perfused. He was alert, oriented to person, place, and time, and conversant. He had no focal deficits, and sensation was intact to light touch in his bilateral upper and lower extremities. To assess strict input and output measurements, a Foley catheter was placed with 200 cc urine output initially.

### Investigations

**Fig. 1:**
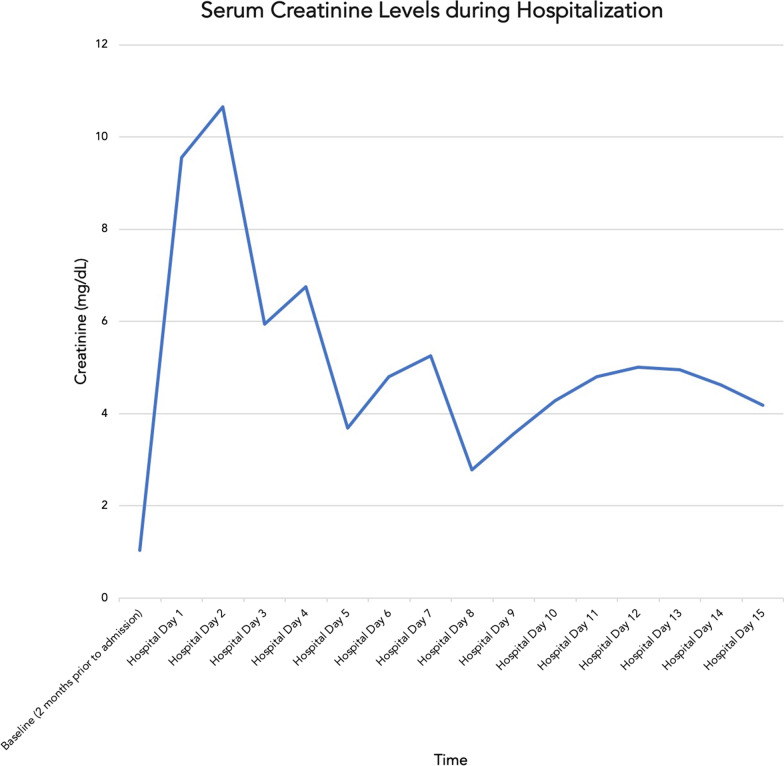
Trend of the creatinine level in the blood during hospitalization

A severe acute respiratory syndrome coronavirus 2 (SARS-CoV-2) ribonucleic acid (RNA) polymerase chain reaction (PCR) via nasopharyngeal swab on admission was positive (Cepheid GeneXpert, N2 cycle threshold of 38.7). Additional laboratory data were notable for decreased sodium of 130 mmol/L (136–144 mmol/L), potassium 3.7 mmol/L (3.3–5.1 mmol/L), chloride of 95 mmol/L (98–107 mmol/L), and bicarbonate of 15 mmol/L (20–30 mmol/L), with elevated anion gap of 20 (7–17). His admission creatinine was 9.55 mg/dL, from a baseline of 1.04 mg/dL (0.40–1.30 mg/dL) when last checked in January 2021 (creatinine trend during hospitalization noted in Fig. 1). His blood urea nitrogen (BUN) was 57 mg/dL (8–23 mg/dL) with estimated glomerular filtration rate (eGFR) of 6 mL per minute per 1.73 m^2^ (> 60 mL per minute per 1.73 m^2^). Creatine kinase level was 521 U/L (11–204 U/L). He had an albumin level of 2.1 g/dL (3.6–4.9 g/dL) and protein level of 5.8 g/dL (6.6–8.7 g/dL). These investigations are noted in Table 1. His white blood cell count was elevated to 15.4 × 1000 cells/µL (4.0–11.0 × 1000 cells/µL), hemoglobin 12.3 g/dL (13.2–17.1 g/dL), MCV (mean corpuscular volume) 78.8 fL (80.0–100.0 fL), platelets 584 × 1000 cells/µL (150–420 × 1000 cells/µL). AST (aspartate aminotransferase) was elevated to 78 U/L (10–35 U/L) and ALT was 34 U/L (9–59 U/L). ALP (alkaline phosphatase) was elevated to 200 U/L (9–122 U/L). D-Dimer was 0.51 FEU (fibrinogen equivalent unit) (≤ 0.67 mg/L FEU). High-sensitivity C-reactive protein was elevated to 114.6 mg/L (≤ 10 mg/L).

**Table 1:** Laboratory investigations

	Value	Reference range
SARS-CoV-2 RNA PCR (nasopharyngeal)	Positive	Negative
Sodium (mmol/L)	130	136–144
Potassium (mmol/L)	3.7	3.3–5.1
Chloride (mmol/L)	95	98–106
Bicarbonate	15	20–30
Anion gap	20	7–17
Creatinine (mg/dL)	0.55	0.40–1.30
Blood urea nitrogen (mg/dL)	57	8–23
Estimated glomerular filtration rate (mL per minute per 1.73 m^2^)	6	> 60
Creatine kinase (U/L)	521	11–204
Albumin (g/dL)	2.1	3.6–4.9
Protein (g/dL)	5.8	6.6–8.7

*RNA* ribonucleic acid, *PCR* polymerase chain reaction

Urinalysis demonstrated turbid, orange urine with a pH of 6, specific gravity of 1.019 with proteinuria (2+ protein), 1+ blood, positive leukocyte esterase, and no nitrite on dipstick testing. Microscopy revealed 0–3 hyaline casts, many white blood cells (unquantified), 0–2 red blood cells per high-power field, and many bacteria. Additionally, urine chemistry demonstrated sodium of 118 mmol/L, potassium of 9 mmol/L, chloride of 82 mmol/L, creatinine of 62 mg/dL, urea nitrogen of 64 mg/dL, random protein of 9.39 g/L, random albumin of 3,782.6 mg/L, with an elevated albumin/creatinine ratio of 6,130.6 mg/g Cr (0–29 mg/g Cr). Urine osmolality was decreased to 298 mOsm/kg (300–900 mOsm/kg). These results are noted in Table 2.

**Table 2:** Urinalysis results

Urine dipstick testing	Value	Reference range
Color	Orange	Yellow
Clarity	Turbid	Clear
pH	6	5.5–7.5
Specific gravity	1.019	1.005–1.030
Protein	2+	Negative to trace
Blood	1+	Negative
Leukocyte esterase	Positive	Negative
Nitrite	Negative	Negative
Glucose	Negative	Negative
Urine microscopy		
Hyaline casts (cells per low-power field)	0–3	0–3
White blood cells (cells per high-power field)	Many	0–5
Red blood cells (cells per high-power field)	0–2	0–2
Bacteria (per high-power field)	Many	None to few
Urine chemistry		
Sodium (mmol/L)	118	Not established
Potassium (mmol/L)	9	Not established
Chloride (mmol/L)	82	Not established
Creatinine (mg/dL)	62	Not established
Urea nitrogen (mg/dL)	64	Not established
Random protein (g/L)	9.39	Not established
Random albumin (mg/L)	3782.6	Not established
Albumin/Cr (mg/g Cr)	6130.5	0–29
Urine osmolality (mOsm/kg)	298	300–900

**Fig. 2:**
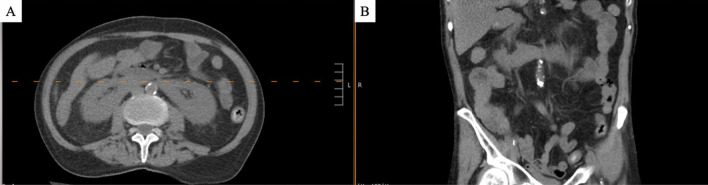
Horseshoe kidney in **A** transverse and **B** coronal view of CT abdomen

He underwent a chest x-ray on admission that showed no acute pathology but revealed chronic underlying emphysematous changes. Computed tomography (CT) of abdomen/pelvis without contrast demonstrated edema or wall thickening of the descending and rectosigmoid colon, suggestive of colitis, as well as a horseshoe kidney (Fig. 2), and a new irregular mass-like scarring lesion and spiculation in the right lower lobe of the lungs and possible ground glass and nodular lesion in the right middle lobe (the latter of which was limited by motion degradation).

For initial management, he received almost 3.5 L of normal saline intravenous fluids administered at 1 L per hour, and was initiated on piperacillin–tazobactam intravenously every 6 hours for two doses. He was subsequently de-escalated to metronidazole 500 mg intravenously once and ceftriaxone 1 g intravenously daily for 3 days (empirically for presumed infectious colitis with potential urinary tract infection) and was transferred to the medical floor as blood pressure responded to intravenous fluids. Antibiotics were changed the next day to piperacillin–tazobactam. When his urine culture eventually grew > 100,000 colony-forming units (CFU)/mL *Escherichia coli*, which was pan-susceptible to all antibiotics tested, he was transitioned to a course of cephalexin 500 mg orally for 4 days. Blood cultures demonstrated no growth.

During the hospitalization, the nephrology service had been consulted and initially felt the etiology of his renal failure was pre-renal in the setting of volume depletion, but progressive renal failure and proteinuria unresponsive to volume resuscitation and repletion with 3.5 L of intravenous fluids raised concern that there was an intrinsic renal pathology. Nephrotoxic medications such as lisinopril 2.5 mg orally were held as was his home rosuvastatin 40 mg daily orally, as creatine kinase was elevated. He had strict monitoring of intake and output as well as frequent monitoring of post-void residuals. There was no evidence of a post-obstructive component of his renal failure. He continued to have poor oral intake during the hospital course. He remained on room air during the hospitalization, aside from episodes of apnea associated with hiccups, which was attributed to vagal nerve dysfunction in setting of recent surgery, and improved with administration of temporary supplemental oxygen at flow rate of 1–2 L per minute via nasal cannula.

On the third day of hospitalization, in the setting of worsening anion gap acidosis, anuria, and worsening renal failure with creatinine reaching 11.5 mg/dL (0.40–1.30 mg/dL), BUN increasing to 72 mg/dL (8–23 mg/dL), and eGFR declining to 4 mL per minute per 1.73 m^2^ (> 60 mL per minute per 1.73 m^2^), he was initiated on intermittent hemodialysis through a Quinton hemodialysis catheter placed into his right internal jugular vein. A workup, including human immunodeficiency virus (HIV) antigen/antibody, hepatitis B surface antigen and hepatitis C antibody testing, was unrevealing. Of note, he did have elevated serum kappa and lambda light chains but no monoclonal spike. On hospital day 3, SARS-CoV-2 nucleocapsid immunoglobulinG (IgG) antibody was positive at 23.4 (< 1).

**Fig. 3:**
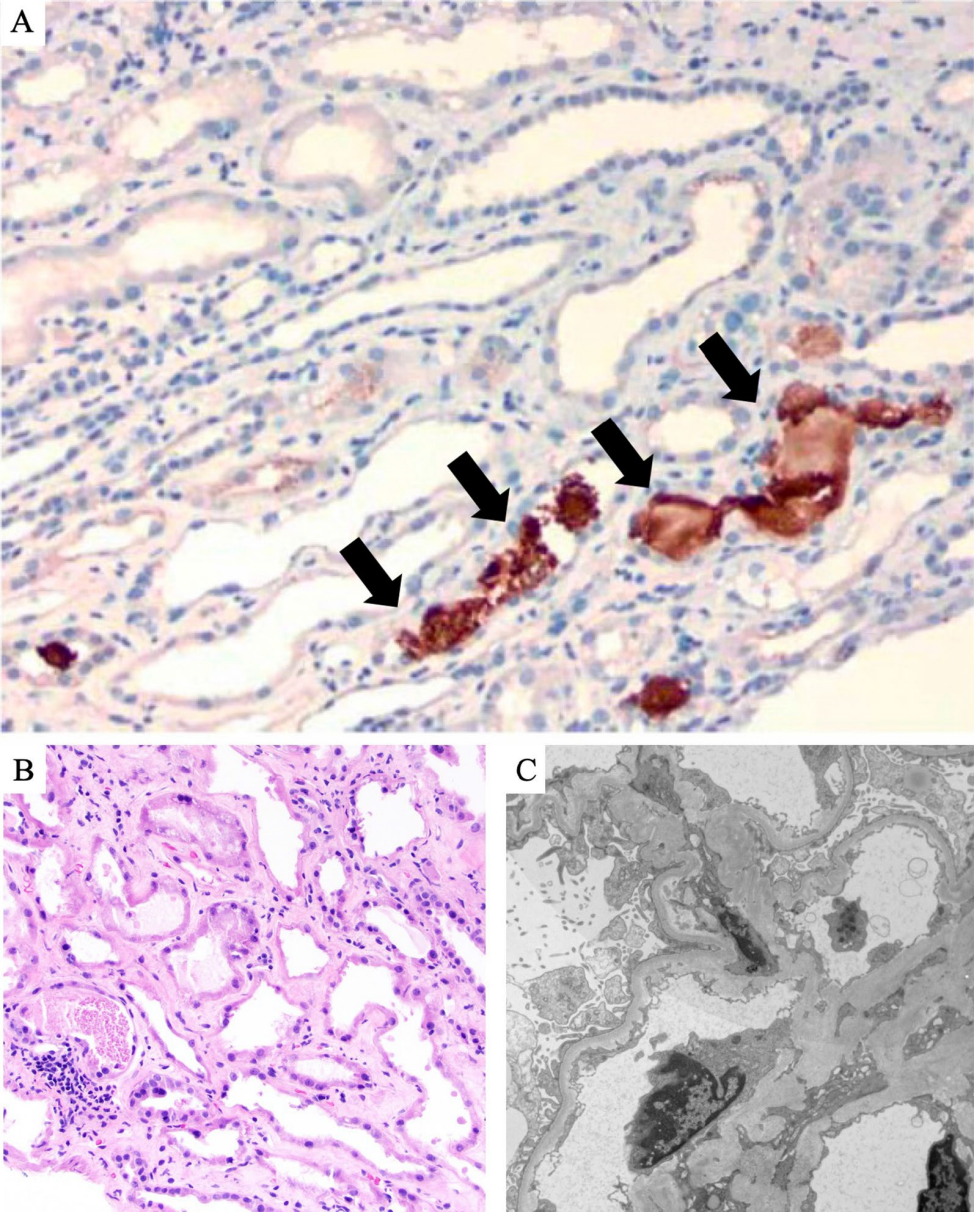
**A** Renal biopsy histopathology under light microscopy with immunohistochemistry strongly positive for myoglobin within casts in tubules (shown by arrows) (cell marque, original magnification, 200×). **B** Granular casts with diffuse acute tubular injury (hematoxylin and eosin; original magnification, 200×). **C** Ultrastructural studies showing corrugation of glomerular basement membranes and segmental foot process effacement (original magnification, 6300×)

On day 4 of hospitalization, he underwent a renal biopsy (Fig. 3). Light microscopy demonstrated a distorted kidney architecture. There was interstitial fibrosis with proportional atrophy involving 30% of the biopsy tissue. There was also a patchy interstitial infiltrate consisting of lymphocytes and plasma cells with rare neutrophils present. The tubules demonstrated acute tubular injury (Fig. 3a). Several intratubular pigmented casts were present. The glomeruli and vessels demonstrated no abnormalities. Ultrastructural findings show corrugation and foot process effacement which suggests hypertension-mediated injury. Immunohistochemistry for myoglobin was positive within casts in tubules (Fig. 3a, b), while hemoglobin A was negative. The kidney biopsy showed negative immunohistochemical staining using an antibody against the SARS-CoV-2 nucleocapsid protein (ThermoFisher, mouse monoclonal antibody clone B46F, dilution 1:200).

Electron microscopy demonstrated patent capillary loops. Glomerular architecture demonstrated corrugation of basement membranes with increase in lamina rara interna (Fig. 3c). No subepithelial deposits and no intramembranous deposits were identified. There was cytoplasmic vacuolization of the podocytes. There was segmental effacement of foot processes (Fig. 3c). There were no subendothelial deposits. Mesangial electron dense deposits were not identified. Electron microscopy did not show presence of viral particles. Tubuloreticular inclusions were not noted. Immunofluorescent testing was not performed in this case in the setting of this being a specimen from a patient with COVID-19.

By day 15 of his hospitalization, his creatinine kinase level had decreased from 521 U/L on admission, to 64 U/L (11–204 U/L); his creatinine downtrended to 4.19 mg/dL toward the end of his hospitalization.

Thus, his acute renal failure was attributed to myoglobin cast nephropathy in the setting of COVID-19-mediated rhabdomyolysis. His proteinuria was attributed to extensive tubular injury.

### Differential diagnosis

The differential diagnoses for rhabdomyolysis and myoglobin cast nephropathy are broad [1–4]. Trauma or excessive muscle breakdown due to overuse such as vigorous exercise can lead to this process. Toxin-mediated rhabdomyolysis due to substances such as cocaine was considered, but this patient did not have history of substance abuse. He also did not report any insect or animal bites. Drug-induced rhabdomyolysis was a possibility since he was on a statin for at least a year. However, the majority of statin-induced myopathies occur within half a year of initiation, though manifestations can appear rarely after this timeframe [5]. There are a number of contributors to rhabdomyolysis, including use of prescribed medications (for example, salicylates, antipsychotics, and benzodiazepines) and nonmedical use of substances (for example, cocaine, cannabis, gamma hydroxybutyrate/gamma butyrolactone, amphetamine, heroin, and alcohol), as well as crush injury or decreased perfusion in the setting of seizures. He did not endorse alcohol use, and none of these other factors was reported by the patient. This was likely not the primary etiology of his rhabdomyolysis, given his acute kidney injury persisted despite his statin medication being held during the hospitalization. In addition, myopathy or myositis due to various etiologies including infection—though relatively rare—must be considered, as was noted with the coincidence of SARS-CoV-2 virus infection and his rhabdomyolysis. Other infectious etiologies that can lead to acute renal injury and rhabdomyolysis include influenza, adenovirus, coxsackievirus, Epstein–Barr virus, cytomegalovirus, Human Immunodeficiency Virus (HIV), herpes simplex virus, and parainfluenza virus [5]. In addition, electrolyte or osmolar abnormalities may be considered as well, though these did not contribute in this patient’s case.

### Treatment

The patient eventually required intermittent hemodialysis for 5 days, until his renal function and urine output gradually recovered. One week later and afterwards, he was producing over a liter of urine daily and no longer required renal replacement therapy and his Quinton catheter was removed.

### Outcome and follow-up

He was discharged to a short-term rehabilitation facility where he stayed for several weeks due to his deconditioned state. Ultimately, the patient’s renal function improved back to his baseline 6 weeks after his initial diagnosis of COVID-19. The patient reported feeling grateful for his improvement in his medical condition. He ultimately received his second dose of the mRNA-1273 COVID-19 vaccine 6 months after the index hospitalization. In the interim, in the setting of his multiple chronic co-morbidities, he was hospitalized multiple times in the interim including for failure to thrive given 30-pound weight loss in the setting of deconditioning, chronic obstructive pulmonary disease exacerbation, atrial fibrillation with rapid ventricular response, and community-acquired pneumonia. At 6 months follow-up, his renal function including creatinine and glomerular filtration rate remained normal. However, this could be confounded by the weight loss. He had no muscle aches or pain.

## Discussion and conclusions

We describe this case report of COVID-19-related acute kidney injury with rhabdomyolysis and notable renal biopsy demonstrating myoglobin cast nephropathy, to add to the limited case series and case reports of COVID-19-related acute kidney injury and rhabdomyolysis.

It is estimated that, for up to one in ten patients with acute renal impairment, rhabdomyolysis may be the underlying etiology [2]. As a result of muscle breakdown, myoglobinemia can occur, leading to myoglobinuria and myoglobin cast nephropathy [2]. In one study that analyzed results of 27,850 renal biopsies performed at a single institution, 214 cases of definitive myoglobin casts were noted, suggesting that its occurrence is not uncommon [2].

Acute kidney injury in patients with COVID-19 may manifest as a range of pathophysiologic entities that may occur singly or in combination, including acute tubular, vascular, glomerular, and interstitial injury [3, 4]. In one study, approximately one of four people hospitalized with COVID-19 experienced acute kidney injury [3]. The pathophysiology of acute kidney injury due to COVID-19 has not yet been fully elucidated. One hypothesized mechanism involves inflammation that is local and systemic, and subsequent cytokine (that is tumor necrosis factor (TNF)-α) release, which leads to injury of the endothelial system and coagulopathy, as well as activation of the renin–angiotensin system [3, 5]. Underlying genetic mechanisms have been reported such as occurrence of COVID-19-associated glomerulopathy in those with certain apolipoprotein L1 genotypes [4]. In this case report, the subject’s renal biopsy sample histopathology had negative SARS-CoV-2 immunostaining. It is worth mentioning that renal failure due to COVID-19 is typically not directly attributable to SARS-CoV-2. Autopsy studies have demonstrated that SARS-CoV-2 viral RNA may be detected in renal tissue through PCR testing, but viral load level is typically lower in the kidney than in the lungs [3]. Even in cases when there is high viral load noted in the kidney, concluding that direct viral cytopathic injury is the etiology of the renal dysfunction is still controversial [4]. Importantly, severe COVID-19-related acute kidney injury may also be due to multiple factors such as hypovolemia, shock, and medication-induced or contrast-related kidney injury that may occur in those with more severe forms of COVID-19 [4].

Other manifestations of renal failure in COVID-19 include hematuria and proteinuria [5]. Patients with COVID-19-induced rhabdomyolysis may classically present with a triad of symptoms: myalgias, tea-colored urine, and muscle weakness [5]. Rhabdomyolysis may present as a late manifestation of COVID-19 occurring weeks to months after detection of the infection [5]. In these patients, lab values such as renal function tests in addition to serum creatine kinase should be monitored, in addition to urine output [5]. Angiotensin-converting enzyme 2 (ACE 2), an important part of the renin–angiotensin–aldosterone system that converts angiotensin II to angiotensin, is an enzyme and receptor, to which the SARS-CoV-2 receptor binding domain binds and enters the cell. Angiotensin-converting enzyme (ACE) 2 receptor are noted on oral mucosal lining, which is thought to contribute to SARS-CoV-2 transmission. ACE 2 is also present predominantly on proximal tubular cells within the kidney, and findings of proteinuria and hematuria have been noted in patients with COVID-19 [6]. With regard to treatment, intravenous fluids may be utilized to treat acute renal failure to equilibrate intravascular volume deficits and correct losses with restoration of urine output [5]. Electrolyte abnormalities should also be monitored and treated appropriately [5]. Moreover, renal replacement therapy may be indicated in those who develop stage III acute kidney injury [5].

While rare case reports and case series of COVID-19-related rhabdomyolysis have previously been described [5, 7, 8], there are limited case reports of COVID-19-related rhabdomyolysis where etiologies have been clearly elucidated following renal biopsies and pathologic examination [7]. Thus, we report this case to highlight that myoglobin cast nephropathy can occur rarely due to COVID-19-mediated rhabdomyolysis and cause severe, but reversible, renal insufficiency.

## Data Availability

The data that support the findings of this study are available but protected under the institutional review board at Yale given the sensitive nature of patient health information, and thus, restrictions apply to the availability of these data, which were used under license for the current study, and so are not publicly available. Data are, however, available from the authors upon reasonable request and with permission from Yale.
